# Manual graphic roadmap for transarterial coil embolization of guttural pouch arteries in horses: A technical note

**DOI:** 10.1111/vsu.70088

**Published:** 2026-02-18

**Authors:** Kathrin Mählmann, Christophorus J. Lischer

**Affiliations:** ^1^ Equine Clinic Freie Universität Berlin Berlin Germany

## Abstract

This technical note describes the use of a manual graphic roadmap (MGR) to assist fluoroscopic guidance during transarterial coil embolization (TACE) for guttural pouch (GP) arterial hemorrhage. Horses underwent MGR‐assisted TACE at the Equine Clinic, Free University of Berlin, between 2012 and 2024. Surgery was performed under general anesthesia with fluoroscopic guidance. Following the initial angiogram, a transparent foil was secured to the fluoroscopy monitor, and an assistant traced the vascular anatomy with a waterproof marker to create the MGR, which then served as a guide for catheter advancement and coil placement. This technique was applied in all TACE procedures performed at the authors' institution. The MGR provided continuous anatomical orientation throughout catheter navigation and coil placement; additional contrast injections were required only to confirm vessel occlusion. We describe a practical approach to support anatomical orientation during TACE in settings without access to fluoroscopy with roadmap technology. Further studies should evaluate cases treated with this technique and assess subjective and objective procedural parameters, including contrast usage, radiation exposure, and overall efficiency, under standardized model conditions.

## 1 INTRODUCTION

Hemorrhage from guttural pouch (GP) arteries is a life‐threatening emergency in horses. It most commonly results from guttural pouch mycosis (GPM), in which fungal invasion erodes the arterial wall. Guttural pouch mycosis primarily affects the internal carotid artery (ICA), but can also effect the external carotid (ECA) or maxillary artery (MA).^1^, ^2^ In some cases, hemorrhage may also occur secondary to aneurysm formation in these vessels.^3^ Immediate vascular occlusion is indicated in cases of epistaxis to prevent exsanguination.^1^

Transarterial coil embolization (TACE) is a well‐established treatment for selective and secure occlusion of affected arteries.^2^, ^4^ Angiography enables identification of anatomical variations and aberrant branches^5^, ^6^ the site of hemorrhage,^4^, ^7^ vascular spasms,^3^ and potential reasons for persistent hemorrhage, such as inadequate recognition of the local vascular anatomy during the initial TACE.^3^

In the originally described TACE technique, the common carotid artery is catheterized, and contrast medium is injected to obtain an angiogram.^4^ After the initial angiogram, the catheter is advanced to the first embolization site. Once the angiographic image disappears, repeated contrast injections are required to navigate to subsequent embolization sites, detect aberrant vessels, estimate vessel diameter, and confirm occlusion.^3^, ^4^

To facilitate continuous orientation during fluoroscopic interventions, modern C‐arm systems are equipped with digital subtraction angiography (DSA) and DSA road mapping (DSA‐RM). In DSA, the precontrast fluoroscopic image is subtracted from the post‐contrast image, removing the soft tissue and bone to display only contrast‐enhanced vessels.^8^ For DSA‐RM the DSA image is superimposed on live fluoroscopy as a static reference to guide real‐time catheter navigation.^9^

At our institution, TACE is the only angiographic intervention performed, and the C‐arm available (Philips BV Endura, 2005, Philips Medical Systems, Best, The Netherlands) does not include the software components for DSA or DSA‐RM. To maintain orientation during catheter advancement, we employed a manual graphic roadmap (MGR) created on a transparent foil with a waterproof marker‐a method previously used anecdotally in human and small animal interventions (personal communications: K. Meisenbacher, Clinic for Vascular and Endovascular Surgery, Heidelberg University Hospital, Germany, March 2024; M. Schneider, Cardiology Service, Small Animal Clinic, Justus Liebig University, Gießen, Germany, April 2024). To our knowledge, this is the first formal description of MGR‐assisted TACE for GP artery occlusion in horses, outlining the step‐by‐step procedure and highlighting its practical advantages and limitations in facilities without DSA‐RM capabilities.

## 2 TECHNIQUE DESCRIPTION

Transarterial coil embolization was performed under general anesthesia with fluoroscopic guidance.^4^

Fluoroscopy was performed with a refurbished C‐arm (Philips BV Endura, Philips Medical Systems). To enable fluoroscopy, the horse's head was positioned toward the caudal end of the surgery table in a semi‐extended position on a padded, radiolucent supporting surface. The x‐ray tube was positioned below the plank. The image intensifier was located as close as possible to the patient to reduce radiation scatter. The proximity of the image intensifier to the patient was limited by the maximum working height of the operating table, which had to allow adequate access to the surgical field.

The fluoroscopic field was adjusted to include the guttural pouch, the basisphenoid and basioccipital bone dorsally, the trachea ventrally, as well as the mandible and the atlas (Figure 1C). Appropriate collimation was applied.

**FIGURE 1:**
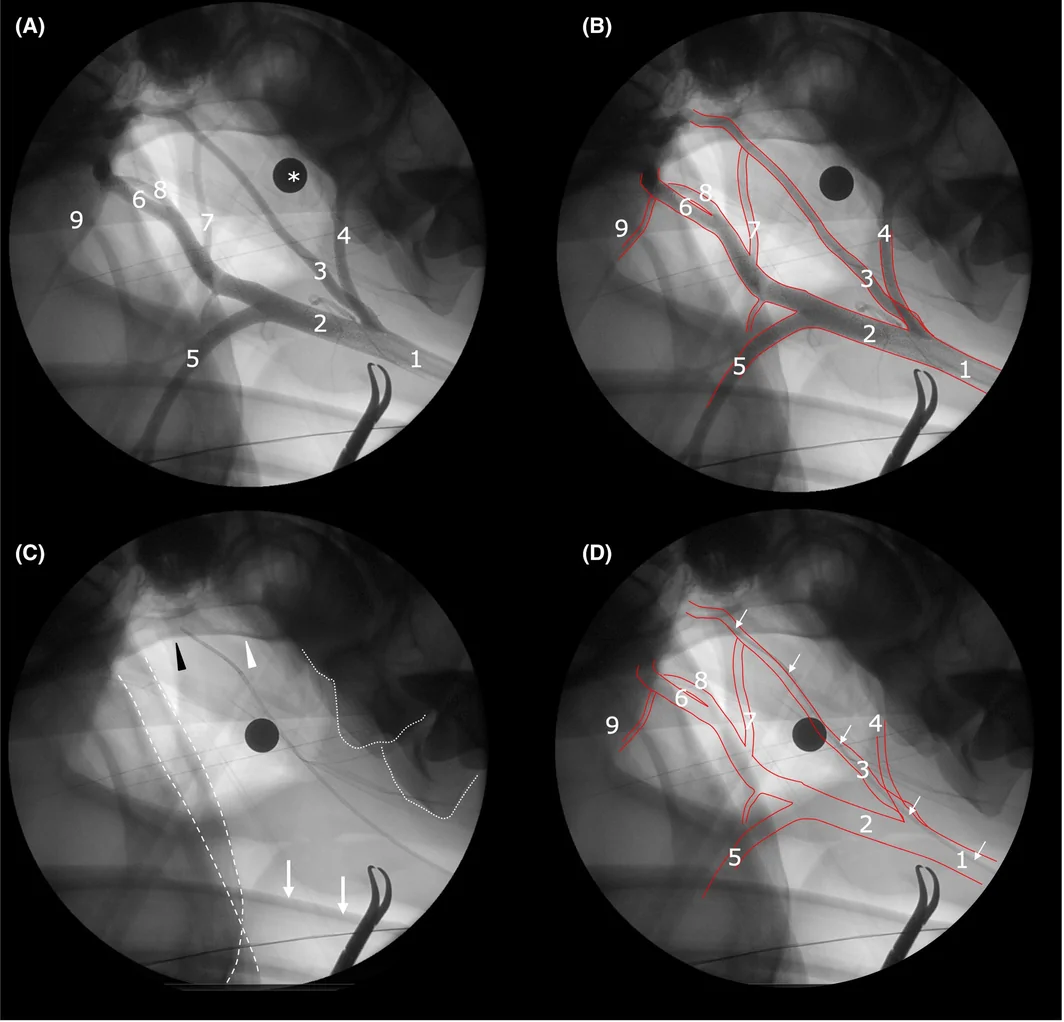
(A) Contrast angiogram; rostral is to the left, caudal to the right, dorsal is at the top of the screen. (B) With the angiogram visible on the screen the outlines of the relevant arteries are traced on the foil with a colored pen (manual graphic roadmap [MGR]). (C) The angiographic catheter is advanced in the internal carotid artery to the site of embolization. The MGR is not shown in this image. Note the radiologic landmarks for orientation: Caudal aspect of the left and right mandibula (dashed line), left and right atlas (dotted line), basisphenoid bone (black arrowhead), basioccipital bone (white arrowhead), endotracheal tube (arrows). (D) The same image as in C is shown with the MGR superimposed with the fluoroscopic picture. The correct advancement of the angiographic catheter (white arrows) can be confirmed throughout the procedure. The MGR was digitally recreated for this illustration. 1: Common carotid artery. 2: External carotid artery. 3: Internal carotid artery. 4: Occipital artery. 5: Linguofacial trunk. 6: Maxillary artery. 7: Caudal auricular artery. 8: Superficial temporal artery. 9. Inferior alveolar artery.

For the MGR, a transparent overhead foil (OHP foil, transparent 210 × 297mm, Pavo Sales B.V., Oss, The Netherlands) was secured to the fluoroscopy monitor with sterilization indicator tape. A standard approach to the common carotid artery was performed at the junction of the proximal and middle thirds of the neck, with careful dissection to preserve the vagosympathetic trunk and recurrent laryngeal nerve.^4^ A 6F introducer system (Flexor Check‐Flo, Cook Medical, Limerick, Ireland) was inserted after puncture of the artery with an entry needle (18 gauge two‐part modified percutaneous entry needle, Cook Medical) directed rostrally. An angiographic catheter (Cook Beacon Tip 5.0 Fr 100 cm, MPA tip, Cook Medical) was primed with heparinized 0.9% saline (10 IU/mL) and introduced over a guidewire (0.035‐inch, 68 180 cm straight‐tip fixed core wire guide, Cook Medical). The catheter was advanced in a rostral direction under fluoroscopic guidance until it appeared proximal to the origin of the internal carotid artery. The guidewire was removed. The initial angiogram was obtained using a double‐flush technique with 10–20 mL of a 1:2 mixture of iopromide (370 mg/mL, Ultravist, Bayer Vital GmbH, Leverkusen, Germany) and heparinized saline, with strict avoidance of air embolism. The catheter was flushed before and after contrast injection with diluted heparinized saline. Any vascular anomalies were noted.

Following the initial angiogram, the last picture was displayed on the screen using the last image hold function and an assistant traced the vessel outlines onto the foil using a colored waterproof pen (Figure 2), creating an accurate representation of the visible branches and angiographic dimensions (Figure 1). In some cases, if the vascular anatomy was not sufficiently delineated, it was necessary to repeat the angiogram and create a new map. However, in the majority of cases, a single angiographic image provided sufficient detail for accurate mapping. The minimum field of view included the ICA, ECA, and MA, along with the origins of the superficial temporal, caudal auricular, inferior alveolar arteries, and linguofacial trunk (Figure 2). The angiogram was stored on the second monitor of the fluoroscope, while real‐time fluoroscopy was displayed on the primary screen, overlaid with the MGR to guide navigation. The catheter was advanced along the MGR to the desired occlusion sites (Figure 1). Embolization of all three arteries was usually performed because preoperative endoscopy was obscured by hemorrhage and therefore inconclusive, endoscopy was not performed due to recent or ongoing bleeding, or because the mycosis was extensive.

**FIGURE 2:**
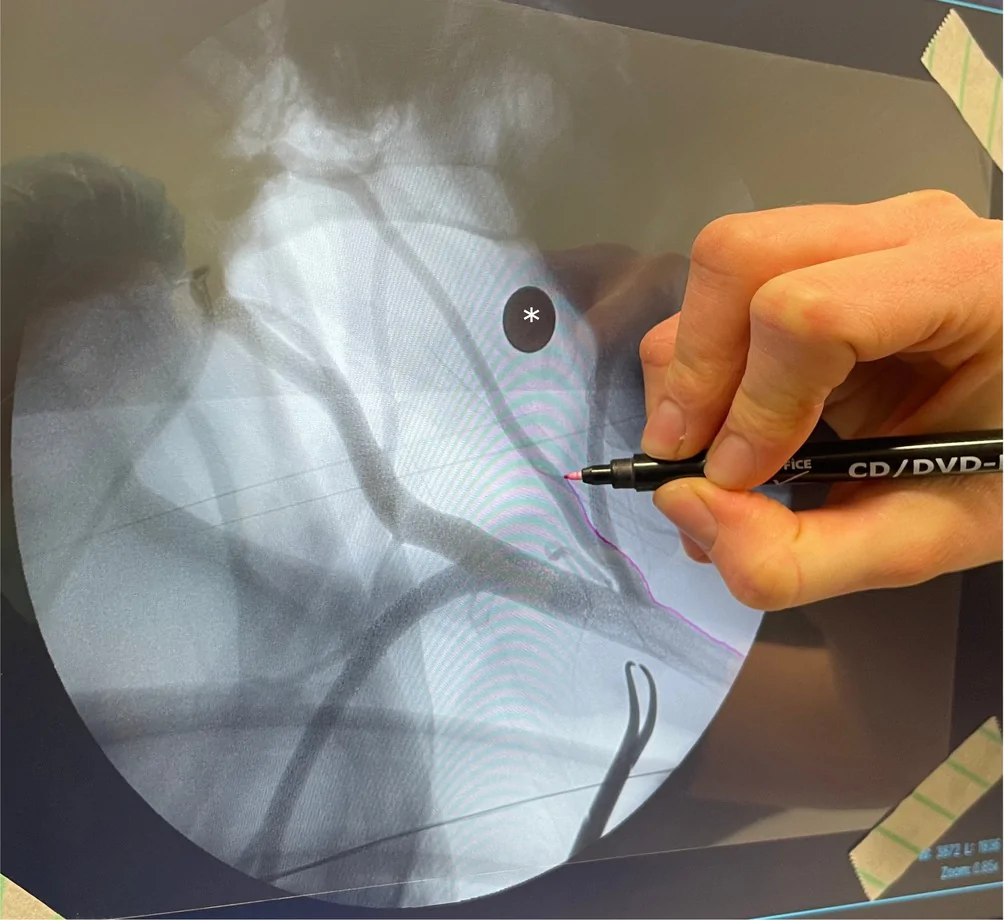
A DIN‐A4 overhead foil is attached to the fluoroscopy monitor with sterilization indicator tape. An unsterile assistant is tracing the internal carotid artery on the initial angiogram using a colored waterproof pen. A metallic sphere (*) measuring 8 mm is placed on the region of the guttural pouch (GP) to account for magnification. Rostral is to the left, caudal to the right, dorsal is at the top of the screen.

Embolization of the internal carotid artery (ICA) was performed as previously described,^4^ with the distal site at the sigmoid flexure, where it is superimposed on the basisphenoid bone, and on the proximal side, midway between the distal embolization point and the origin of the ICA from the common carotid artery.

The embolization site for the maxillary artery (MA) was located between the origin of the superficial temporal artery proximally and the origins of the infraorbital, buccal, and inferior alveolar arteries distally. Embolization of the external carotid artery (ECA) was localized between the origin of the caudal auricular artery and the origin of the linguofacial trunk.

Coil size was determined by comparing vessel diameter on the MGR or angiogram to the projection of a sterile 8 mm stainless steel sphere placed over the GP region to correct for magnification, using the equation: Real vessel diameter = (real sphere diameter × measured vessel diameter)/measured sphere diameter. Coils (Cook Medical, MReye embolization coil) were inserted at the reported occlusion sites^4^ using a 0.035‐inch, 180 cm straight‐tip fixed core wire guide (Cook Medical). A control angiogram was performed for each embolization site by injecting 5 mL of contrast^4^ under gentle, steady pressure to confirm complete occlusion.

After the procedure, the introducer system was removed and the arteriotomy closed with a preplaced cruciate suture of 4–0 poliglecaprone 25. Muscle layers and skin were closed with a simple continuous pattern, and a stent bandage was sutured in place.

The MGR technique was applied for all TACE procedures performed at the authors' institution from 2012 to 2024. No additional contrast injections were used to detect vessel bifurcations, navigate curved sections, or determine coil position relative to other vessels but only to confirm final occlusion.

## 3 DISCUSSION

This technical note describes the use of a MGR to guide fluoroscopy during TACE for occluding GP arteries in horses under general anesthesia in the absence of modern fluoroscopy systems. A MGR generated from the initial angiogram served as a continuous anatomical reference. In the authors' experience, it enhanced spatial orientation and enabled accurate catheter guidance and coil deployment throughout the procedure. Decoupling catheter navigation from repeated contrast injections also simplifies equipment handling and may reduce procedural complexity.

Although preparation of the angiographic roadmap by an assistant requires a few minutes, it can ultimately shorten the overall procedural time. By providing a continuous visual guide for catheter navigation, the MGR‐assisted approach minimizes the need for repeated catheter repositioning and additional angiographic acquisitions, allowing the operator to reach target vessels more efficiently compared with TACE performed without a roadmap.^4^ This, in turn, may reduce overall radiation exposure. Lowering the use of contrast medium and radiation may further decrease the risks associated with arterial angiography in humans, including cardiovascular reactions to contrast agents,^10^, ^11^ neurologic complications^12^, ^13^ and the deterministic and stochastic effects of ionizing radiation.^14^

While these advantages are supported by procedural experience, no direct comparison to non‐MGR cases was possible, as all TACE procedures at our clinic employed MGR. While comparisons with other studies could help to validate these observations, no published data report total contrast volume, number of injections, or radiation exposure for TACE in horses.

The success of the MGR relies on the surgery assistant's accuracy in tracing the vascular anatomy, whereas DSA‐RM avoids drawing inaccuracies by preserving the exact vessel configuration and proportions. In both techniques, only contrast‐filled vessels can be visualized, and the angiogram must be repeated if the vascular anatomy is not clearly delineated. Both techniques require the patient to remain immobile; movement necessitates roadmap recreation. While DSA has been used in horses with GPM to identify vascular pathologies and abnormalities,^4^, ^5^ or to confirm arterial occlusion,^4^ DSA‐RM has not been reported for TACE in horses. At the authors' institution, DSA‐RM is not available, which prevents direct comparison to MGR. To date, no experimental studies have evaluated MGR accuracy relative to DSA‐RM.

In complex vascular interventions such as endovascular aneurysm repair in humans, three‐dimensional orientation is increasingly achieved using image fusion.^15^ For this purpose, preprocedural datasets, such as arterial‐phase computed tomography (CT) or magnetic resonance imaging, are acquired and fused with intraprocedural fluoroscopy or C‐arm cone‐beam CT.^15^ As CT and cone‐beam CT become more accessible, these may benefit TACE in horses, particularly for detecting anatomical variants and localizing lesions, though costs remain prohibitive for most equine facilities.

Future research using controlled settings, such as post‐mortem perfused equine models or three‐dimensional (3D)‐printed vascular reproductions, could enable standardized assessments. This would allow systematic comparison of procedural time, number of contrast injections, contrast volume, accuracy, and radiation exposure among TACE, MGR‐assisted TACE, and DSA‐RM. Retrospective evaluation of clinical outcomes in horses treated with MGR‐assisted TACE is also warranted.

This technical note introduces MGR as a practical method to enhance fluoroscopic guidance during TACE in facilities without modern fluoroscopy systems.

## AUTHOR CONTRIBUTIONS

Mählmann K, DECVS (Large Animal): Prepared the manuscript and illustrations. Lischer C, DECVS (Large Animal): Contributed to the design of the note, and provided scientific, in‐line editing of the manuscript. All authors provided a critical review of the manuscript and endorse the final version. All authors are aware of their respective contributions and have confidence in the integrity of all contributions.

## CONFLICT OF INTEREST STATEMENT

The authors declare no conflict of interest related to this report.
